# Ameliorative Effect of Gallic Acid on Cyclophosphamide-Induced Oxidative Injury and Hepatic Dysfunction in Rats

**DOI:** 10.3390/medsci3030078

**Published:** 2015-09-08

**Authors:** Ebenezer Tunde Olayinka, Ayokanmi Ore, Olaniyi Solomon Ola, Oluwatobi Adewumi Adeyemo

**Affiliations:** Biochemistry Unit, Department of Chemical Sciences, Ajayi Crowther University, PMB 1066, Oyo, Oyo State 211213, Nigeria; E-Mails: oreayokanmi@gmail.com (A.O.); olaolaniyis@yahoo.com (O.S.O.); dewumyt@gmail.com (O.A.A.)

**Keywords:** cyclophosphamide, gallic acid, oxidative injury, hepatic dysfunction

## Abstract

Cyclophosphamide (CP), a bifunctional alkylating agent used in chemotherapy has been reported to induce organ toxicity mediated by generation of reactive oxygen species and oxidative stress. Gallic acid (GA), a phenolic substance, is a natural antioxidant with proven free radical scavenging activity and offers protection against oxidative damage. This research study was designed to investigate the ameliorative effect of GA against CP-induced toxicity in rats. Twenty-five male Wistar rats (180–200 g) were randomized into five treatment groups: (A) control, (B) CP, 2 mg/kg body weight (b.w.), (C) pre-treatment with GA (20 mg/kg b.w.) for seven days followed by CP (2 mg/kg b.w.) for seven days, (D) co-treatment with GA (20 mg/kg b.w) and CP (2 mg/kg b.w.) for seven days, and (E) GA (20 mg/kg b.w.) for seven days. CP induced marked renal and hepatic damages as plasma levels of urea, creatinine, bilirubin and activities of AST, ALT, ALP and GGT were significantly elevated (*p <* 0.05) in the CP-treated group relative to control. In addition, hepatic levels of GSH, vitamin C and activities of SOD, catalase and GST significantly reduced in the CP-treated group when compared with control. This was accompanied with a significant increase in hepatic lipid peroxidation. The restoration of the markers of renal and hepatic damages as well as antioxidant indices and lipid peroxidation by pre- and co-treatment with GA clearly shows that GA offers ameliorative effect by scavenging the reactive oxygen species generated by CP. This protective effect may be attributed to the antioxidant property of gllic acid.

## 1. Introduction

**Figure 1:**
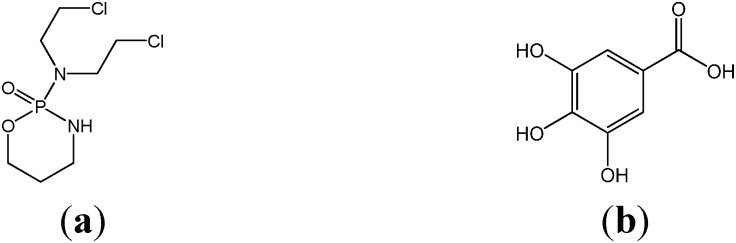
Chemical structure of cyclophosphamide (**a**), and gallic acid (**b**).

Cyclophosphamide (CP), Figure 1a, is a synthetic alkylating agent chemically related to the nitrogen mustards [1] widely used as an anticancer and immunosuppressive drug [2,3] and in the treatment of nephrotic syndrome [4,5]. It is effective against a wide spectrum of malignancies, such as, leukemia, lymphoma, breast, lung, prostate, and ovarian cancers [6,7]. CP is an inactive cytostatic alkylating agent, which is metabolized into active metabolites mainly in the liver. Phospharamide mustard and acrolein are its two active metabolites produced by the liver microsomal enzymes [8,9]. Many anticancer drugs are known for the generation of Reactive Oxygen Species (ROS) in cancer cells [10] and these ROS generated lead to oxidative damage in the cell [11]. During bioactivation of CP, reactive oxygen species are also formed, which can modify the components of both healthy and neoplastic cell leading to decreased antioxidative capacity [12]. CP’s antineoplastic effects are associated with phosparamide mustard, while acrolein is linked with its toxic side effects [13]. CP has been reported to produce genotoxicity and oxidative stress in mice [14] and early lung injury in rats [15]. Numerous studies have shown that CP exposure can disrupt the redox balance of tissues and that these biochemical and physiological disturbances resulted from oxidative stress may be implicated in disorders like hemorrhagic cystitis, testicular gametogenic and androgenic disorders, liver and kidney disorders, inhibition of ovarian steroidogenesis, *etc.* [16,17,18,19,20,21]. The cytotoxic effects of CP and other chemotherapeutic drugs result in part from their interaction with DNA leading to defective DNA, abnormal cell function and cell death [22]. The toxicities associated with CP have led to an increasing search for effective model compounds that could protect against its induced organ toxicity [17,19,20]. Several studies suggest that antioxidant supplementation can influence the response to chemotherapy as well as the development of adverse side effects that result from treatment with antineoplastic agents [23]. The potential role of dietary antioxidants, such as ascorbic acid, tocopherol, β-carotene, *etc.*, to reduce the activity of free radical-induced reactions has drawn increasing attention [24]. Gallic acid (GA), a polyhydroxyphenolic compound (3,4,5-trihydroxybenzoic acid, Figure 1b), is a naturally occurring plant phenol present in nutgalls, green tea, grapes, red wine, hops, oak bark and other plants [25,26,27,28]. GA is a powerful and natural antioxidant and possesses a number of biological and pharmacological activities including scavenging of free radicals, anti-inflammatory and antiapoptotic [29,30,31]. Other effects include protection against CCl_4_-induced hepatotoxicity [32], Lindane-induced hepatic and renal toxicity [33], and doxorubicin-induced myocardial toxicity [34]. This study is therefore aimed at evaluating the protective effect of gallic acid on cyclophosphamide induced toxicity and oxidative stress in rats.

## 2. Materials and Methods

### 2.1. Chemicals and Reagents

Cyclophosphamide tablet is a product of West Coast Pharmaceutical Works Ltd, Gota, Ahmedabad, India. Glutathione, 1-chloro-2,4-dinitrobenzene (CDNB), 5,5-dithiobis-2-nitrobenzoic acid (DTNB), epinephrine, and hydrogen peroxide (H_2_O_2_) were all purchased from Sigma Chemical Company (London, UK). Kits for alanine aminotransferase (ALT), aspartate aminotransferase (AST), alkaline phosphatase (ALP), gamma glutamyl transferase (GGT), Urea, Creatinine and total Bilirubin were obtained from Randox laboratories ltd. (Antrim, UK). All other reagents used were of analytical grade and of highest purity.

### 2.2. Animals and Treatments

Male rats (Wistar strain) weighing between 180–200 g were used in this study. The rats were bred and housed in the animal house of the Department of Chemical Sciences, Ajayi Crowther University, Oyo, Nigeria. They were kept in wire-meshed cages at room temperature and under controlled light cycle (12 h light:dark). They were fed with commercial rat chow (Ladokun feeds, Ibadan, Nigeria) and water *ad libitum*. All experiments were conducted without anesthesia and protocol conforms to the guidelines of the National Institute of Health for laboratory animal care and use (National Research Council, Washington, USA) [35].

### 2.3. Experimental Design

This study employed a simple randomized design. Twenty-five male albino rats (Wistar strain) were randomized into five groups of 5 rats each, using a randomization table. All animals were the same age. The randomization was done by the distribution of rats in separate cages. Animals of similar weight were gathered in one group. The groupings were determined by the alkylating agent and antioxidant used. Sample size was determined according to “resource equation method” described by Charan and Kantharia [36]. Group A animals were used as control and received distilled water. Group B animals were treated with cyclophosphamide (CP) only (equivalent to 2 mg/kg body weight of cyclophosphamide). The Group C animals were pre-treated with 20 mg/kg body weight gallic acid (GA) for seven days, after which administration of CP commences for another seven days, while Group D animals were co-treated with daily administration of GA (equivalent to 20 mg/kg body weight of gallic acid) and CP (2 mg/kg b.w.) for seven days. Group E animals received daily administration of gallic acid only for seven days. One milliliter each of the prepared CP and GA solution was administered orally to the animals in the different groups using an oral cannula once daily for a period of seven days (the normal duration of therapy in humans). The dose for CP was arrived at based on recommended adult dose for leukemia treatment while the dose for GA was decided from information available in literature [37]. The animals were euthanized 24 h after the last treatment.

### 2.4. Collection of Blood Samples for Plasma Preparation and Animal Sacrifice

Blood was collected from the retro orbital plexus of the animals into heparinized tubes, and the rats were sacrificed by cervical dislocation. Plasma was prepared by centrifuging blood samples for 5 min at 4000 rpm using a bench centrifuge (Analytica, Athens, Greece). The clear supernatant was used for the estimation of urea, creatinine, bilirubin and enzymes.

### 2.5. Preparation of Cytosolic Fractions

The liver excised from rat, blotted of blood stains, and rinsed in 1.15% KCl, was homogenized in 4 volumes of ice-cold 0.01 M potassium phosphate buffer (pH 7.4). The homogenates were centrifuged at 12,500 *g* in a refrigerated centrifuge (Eppendorf, Stevenage, UK) for 15 min at 4 °C and the supernatants, termed as the post mitochondrial fractions (PMF), were used for enzyme assays.

### 2.6. Renal and Liver Functions Test

Plasma creatinine, urea, and bilirubin determination was done using Randox diagnostic kits. Methods for creatinine assays are based on colorimetric alkaline picrate methods [38] with creatinine-picrate complex measured at 492 nm. The urea determination method was based on the Fenton reaction [39], with the Diazine chromogen formed being absorbed strongly at 540 nm. The dimethyl sulfoxide method by Tietz *et al.* [39] was used for bilirubin determination. The dimethyl sulfoxide forms a colored compound with maximum absorption at 550 nm.

### 2.7. Determination of Plasma AST, ALT, ALP, and GGT Activities

Plasma AST, ALT, ALP, and GGT activities were determined using Randox diagnostic kits. Determination of AST and ALT activities was based on the principle described by Tietz *et al.* [39]. AST was measured by monitoring the concentration of oxaloacetate hydrazone formed with 2,4-dinitrophenylhydrazine at 546 nm, and ALT was measured by monitoring the concentration of pyruvate hydrazone formed with 2,4-dinitrophenylhydrazine at 546 nm. ALP was determined in accordance with the principles of Tietz [40]. The *p*-nitrophenol formed by the hydrolysis of *p*-nitrophenyl phosphate confers yellowish color on the reaction mixture and its intensity can be monitored at 405 nm to give a measure of enzyme activity. GGT activity was measured based on a modification of the method described by Horder *et al.* [41] using Abbott diagnostic kit (Abbott Laboratories, North Chicago, Illinois, USA).

### 2.8. Assay of Nonenzymatic Antioxidants and Lipid Peroxidation

Hepatic vitamin C was determined according to the method of Erel *et al.* [42] using dinitro phenyl hydrazine (DNPH), while hepatic glutathione was determined according to the method of Jollow *et al.* [43]. The chromophoric product resulting from the reaction of Ellman’s reagent with the reduced glutathione, 2-nitro-5-thiobenzoic acid possesses a molar absorption at 412 nm, which was read in a spectrophotometer. Reduced GSH is proportional to the absorbance at 412 nm. The extent of lipid peroxidation (LPO) was estimated by the method of Varshney and Kale [44]; the method involved the reaction between malondialdehyde (MDA) and thiobarbituric acid to yield a stable pink chromophore with maximum absorption at 532 nm.

### 2.9. Determination of Antioxidant Enzymes

The procedure of Misra and Fridovich [45] as described by Magwere *et al.* [46] was used for the determination of hepatic superoxide dismutase (SOD) activity by measuring the inhibition of autooxidation of epinephrine at pH 10.2 and 30 °C. SOD activity was expressed in U/mg protein. Hepatic catalase activity was determined according to the method of Sinha [47] by measuring the reduction of dichromate in acetic acid to chromic acetate at 570 nm. Catalase activity was expressed as µmol H_2_O_2_ consumed/min/mg protein. Hepatic glutathione *S*-transferase (GST) activity was determined by the method described by Habig *et al.* [48] using 1-chloro-2,4-dinitrobenzene (CDNB) as substrate. GST activity was expressed in µmol/min/mg protein.

### 2.10. Protein Determination

Protein content of plasma and all fractions was estimated by the method of Lowry *et al.* [49] using bovine serum albumin as standard.

### 2.11. Statistical Analysis

Results were expressed as mean of 5 replicates ±SD. Data obtained were subjected to one way Analysis of Variance (ANOVA) and complemented with Duncan’s multiple range test using Stat Pac Statistical Software (Systat Software Inc. San Jose, CA, USA). Statement of statistical significance was based on *p* < 0.05.

## 3. Results

### 3.1. Protective Effects of Gallic Acid on Cyclophosphamide Induced Changes in the Levels of Plasma Creatinine, Urea and Bilirubin in Rats

Table 1 shows the Protective effects of gallic acid on cyclophosphamide induced changes in the levels of plasma creatinine, urea and bilirubin in rats. Plasma creatinine, urea, and bilirubin were significantly increased (*p* < 0.05) in the CP treated groups by 139%, 24%, and 72%, respectively, relative to the control. Pre-treatment and co-treatment with the GA significantly protected against the increase in the levels of urea, creatinine and bilirubin when compared CP-treated animals.

**Table 1:** Protective effects of gallic acid on cyclophosphamide induced changes in the levels of plasma creatinine, urea and bilirubin in rats.

Treatment	Creatinine (mg/dL)	Urea (mg/dL)	Bilirubin (mg/dL)
**Control**	0.98 ± 0.13	51.4 ± 3.2	0.36 ± 0.03
**CP**	2.34 ± 0.11 (139%) *	63.8 ± 3.7 * (24%)	0.62 ± 0.02 (72.2%) *
**CP + GA (Pre-treated)**	1.56 ± 0.1 *^,a^	54.2 ± 2.4 *^,a^	0.55 ± 0.02 *^,a^
**CP + GA (Co-treated)**	1.48 ± 0.2 *^,a^	53.6 ± 2.9 *^,a^	0.52 ± 0.02 *^,a^
**GA**	1.0 ± 0.1	50.3 ± 2.8 *	0.38 ± 0.01

CP: cyclophosphamide (2 mg/kg body weight); GA: gallic acid (20 mg/kg body weight); Data are expressed as mean ±SD for five rats in each group; * Significantly different from the control (*p* < 0.05); ^a^ Significantly different from cyclophosphamide group; Values in parenthesis represent percentage (%) increase.

### 3.2. Protective Effects of Gallic Acid on Cyclophosphamide-Induced Changes in the Activities of Plasma Alanine Aminotransferase (ALT), Aspartate Aminotransferase (AST), Alkaline Phosphatase (ALP), and Gamma Glutamyl Transferase (GGT) in Rats

The Protective effects of GA on CP induced changes in the activities of Plasma ALT, AST, ALP and GGT in rats is represented in Table 2. Administration of CP significantly increased the plasma activities of ALT, AST, ALP and GGT by 106%, 31%, 190% and 142%, respectively, when compared to the control. However, pre-treatment and co-treatment with the GA significantly attenuated the CP-induced increase in plasma ALT, AST, ALP and GGT relative to CP-treated rats.

**Table 2:** Protective effects of gallic acid on cyclophosphamide induced changes in the activities of plasma alanine aminotransferase (ALT), aspartate aminotransferase (AST), alkaline phosphatase (ALP), and gamma glutamyl transferase (GGT) in rats.

Treatment	ALT (U/L)	AST (U/L)	ALP (U/L)	GGT (U/L)
**Control**	21.1 ± 1.4	174 ± 2.30	257 ± 2.41	7.1 ± 0.3
**CP**	43.4 ± 2.5 (106%) *	228 ± 8.7 (31%) *	746.4 ± 8.05 (190%) *	17.2 ± 1.3 (142%) *
**CP + GA (Pre-treated)**	30.2 ± 1.8 *^,a^	192.8 ± 5.9*^,a^	444 ± 18.2 *^,a^	11.4 ± 0.84 *^,a^
**CP + GA (Co-treated)**	29.1 ± 1.6 *^,a^	188.4 ± 7.4 *^,a^	404.4 ± 8.26 *^,a^	11.2 ± 1.3 *^,a^
**GA**	20.3 ± 2.6	177.6 ± 5.4	263.6 ± 2.61	6.8 ± 0.9

CP: cyclophosphamide (2 mg/kg body weight); GA: gallic acid (20 mg/kg body weight); Data are expressed as mean ±SD for five rats in each group; * Significantly different from the control (*p* < 0.05); ^a^ Significantly different from cyclophosphamide group; Values in parenthesis represent percentage (%) increase.

### 3.3. Protective Effects of Gallic Acid on Cyclophosphamide-Induced Changes in the Activities of Hepatic Enzymatic Antioxidants in Rats

**Figure 2:**
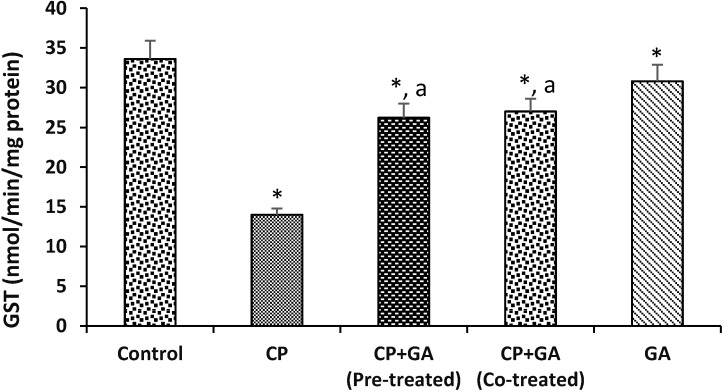
Protective effects of gallic acid on cyclophosphamide induced changes in the activity of hepatic glutathione-*S*-transferase (GST) in rats. CP = cyclophosphamide (2 mg/kg body weight); GA = gallic acid (20 mg/kg body weight); Data are expressed as mean ±SD for five rats in each group; * Significantly different from the control (*p* < 0.05); ^a^ Significantly different from cyclophosphamide group.

Protective effects of GA on CP-induced changes in the activities of hepatic Superoxide dismutase (SOD) and Catalase in rats are shown in Table 3. Hepatic SOD and Catalase activities were reduced significantly by 51% and 52% in CP-treated animals as compared to control. Pre-treatment and co-treatment with GA significantly ameliorated against the decrease in SOD and Catalase activities when compared to CP-treated animals. In addition, Figure 2 shows the protective effects of GA on CP-induced changes in the activity of hepatic GST in rats. The activity of GST in the liver of animals treated with CP showed a decrease of 55% as compared to control group. However, pre-treatment and co-treatment with the GA was able to protect significantly when compared to CP-treated animals.

**Table 3:** Protective effects of gallic acid on cyclophosphamide induced changes in the activities of hepatic superoxide dismutase (SOD) and catalase (CAT) in rats.

Treatment	SOD (units)	CAT (µmol H_2_O_2_ consumed/min/mg protein)
**Control**	4.8 ± 0.3	0.23 ± 0.03
**CP**	2.34 ± 0.2 (51%) *	0.11 ± 0.01 (52%) *
**CP + GA (Pre-treated)**	4.06 ± 0.2 *^,a^	0.17 ± 0.01 *^,a^
**CP + GA (Co-treated)**	3.9 ± 0.1 *^,a^	0.17 ± 0.02 *^,a^
**GA**	4.65 ± 0.1	0.22 ± 0.01

CP: cyclophosphamide (2 mg/kg body weight); GA: gallic acid (20 mg/kg body weight); Data are expressed as mean ±SD for five rats in each group; * Significantly different from the control (*p* < 0.05); ^a^ Significantly different from cyclophosphamide group; Values in parenthesis represent percentage (%) decrease.

### 3.4. Protective Effects of Gallic Acid on Cyclophosphamide Induced Changes in the Levels of Hepatic Nonenzymatic Antioxidants and Lipid Peroxidation

**Figure 3:**
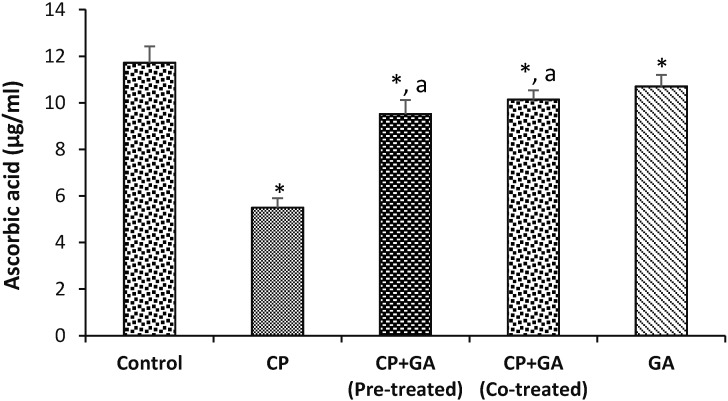
Protective effects of gallic acid on cyclophosphamide induced changes in the levels of hepatic ascorbic acid in rats. CP = cyclophosphamide (2 mg/kg body weight); GA = gallic acid (20 mg/kg body weight); Data are expressed as mean ±SD for five rats in each group; * Significantly different from the control (*p* < 0.05); ^a^ Significantly different from cyclophosphamide group.

**Figure 4:**
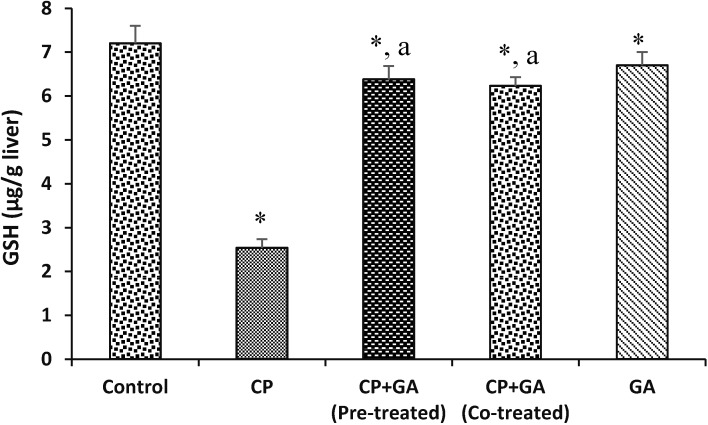
Protective effects of gallic acid on cyclophosphamide induced changes in the levels of hepatic reduced glutathione (GSH) concentration in rats. CP = cyclophosphamide (2 mg/kg body weight); GA = gallic acid (20 mg/kg body weight); Data are expressed as mean ±SD for five rats in each group; * Significantly different from the control (*p* < 0.05); ^a^ Significantly different from cyclophosphamide group.

**Figure 5:**
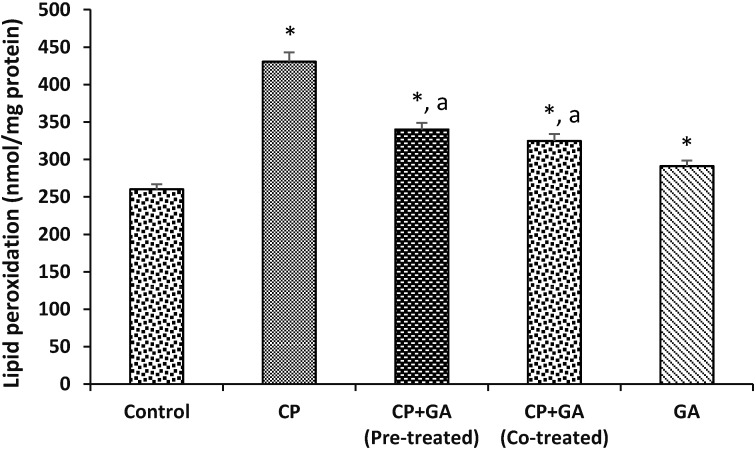
Protective effects of gallic acid on cyclophosphamide induced changes in the levels of hepatic Lipid peroxidation in rats. CP = cyclophosphamide (2 mg/kg body weight); GA = gallic acid (20 mg/kg body weight); Data are expressed as mean ±SD for five rats in each group; * Significantly different from the control (*p* < 0.05); ^a^ Significantly different from cyclophosphamide group.

Figure 3 and Figure 4 present the protective effects of GA on CP-induced changes in the levels of hepatic ascorbic acid (AA) and GSH in rats. The hepatic AA and GSH levels were significantly decreased by 53% and 65%, respectively, in the treated groups when compared with the control. The protective effects of GA on CP-induced changes in hepatic Lipid peroxidation (LPO) in rats are shown in Figure 5. The level of LPO in the liver of the animals treated with CP increased significantly (*p* < 0.05) by 66% as compared to control group. Pre-treatment and co-treatment with the GA significantly protects against the decrease in hepatic AA and GSH and increase in LPO relative to CP-treated group.

## 4. Discussion

Cyclophosphamide (CP) is an inactive cytostatic alkylating agent that is metabolized into active metabolites, phosphoramide mustard and acrolein by liver microsomal enzyme [50]. It has been reported that during bioactivation of cyclophosphamide, reactive oxygen species are also formed, which can modify the components of both healthy and neoplastic cell leading to decreased antioxidative capacity [51].

Recently, the search of potential compounds of plant origin that has ameliorative capacity to minimize chemotherapeutic toxicity to normal cells without affecting their antineoplastic activity has increased [52]. Gallic acid (GA, 3,4,5-trihydroxybenzoic acid), a polyhydroxy phenolic compound is a naturally occurring plant phenol [53] known to possess strong antioxidant, anti-inflammatory and antiapoptotic properties [29,30,31].

The present investigation was carried out to evaluate the protective potential of gallic acid (GA) against oxidative stress-mediated hepatocellular toxicity induced by CP in rats. The activities of the liver enzymes ALT, AST, ALP and GGT in the plasma are reliable markers for the assessment of hepatic injury [54]. Elevated plasma levels of these enzymes by CP are indicative of cellular damages and loss of functional integrity of hepatocyte membrane leading to their leakage into the serum or plasma [55,56,57]. AST is an enzyme abundant in the cytoplasm and mitochondria of liver and also present in the heart, skeletal muscle and brain. ALT is hepatospecific enzyme principally found in the cytoplasm [58,59]. ALP and GGT are associated with the cell membrane and their increase in the plasma is an indication of impairment of intrahepatic and extra-hepatic bile flow (cholestasis), hepatobiliary injury and overproduction or leakage of ALP and GGT [60,61]. From the result of this study, pre-treatment and co-treatment with GA restored the activities of ALT, AST, ALP and GGT. The hepatoprotective effects of GA in this study confirm the findings from previous studies [32,62].

Urea and creatinine are the waste products of metabolism that are freely filtered by the glomeruli of the kidneys [63]. Their concentrations in the blood plasma are used for the screening of renal or cardiovascular disorders [64]. Significant increase in plasma levels of creatinine and urea by CP is an indication of abnormal renal function which might be due to intrinsic renal lesions that are observed only with marked damage to functioning nephrons [65,66]. Moreover, elevated level of bilirubin observed in CP treated animal may be linked with intra and extra cellular hemolysis. GA ameliorated the CP-induced hepatic and renal injuries. These reductions observed in the levels of the renal and hepatic markers are an indication of protective activities of GA against active toxic metabolites of CP.

Reports have shown that there is a link between oxidative stress and liver tissue injuries [67,68]. Alkylating agents including CP are known to have pro-oxidant characters, generating reactive oxygen species (ROS) resulting in depletion of cellular detoxifying thiols and antioxidant enzymes [69,70]. In this study, CP caused a significant reduction in hepatic SOD, catalase (CAT), GST, ascorbic acid (AA) and GSH with a concomitant increase in lipid peroxidation. SOD catalyzes the removal of superoxide ion (O_2_^−^) by converting it to hydrogen peroxide (H_2_O_2_), which in turn could be rapidly converted to water and oxygen by CAT [71]. A reduction in the activities of SOD and CAT by CP observed in this study might render the liver more susceptible to attack by O_2_^−^ and H_2_O_2_ and hydroxyl radical-induced oxidative stress.

The levels of AA and GSH provide a measure of the cellular redox status. Both AA and GSH are free radical scavengers in cells and are often the first line of defense against oxidation [72,73]. AA is a water-soluble vitamin with biological activity to scavenge free radicals by reacting with oxygen radical to generate a less radical, semialdehyde ascorbate. GSH system is a key component of the overall defense system that has the capacity of detoxifying both endogenous and exogenous toxic substances and also in regenerating vitamin C and E to their active forms [74,75]. GST is an enzyme involved primarily in the detoxification of highly reactive electrophiles such as drugs by combining with GSH as conjugating agent [76]. The decrease observed in the levels of AA and GSH as well as activity of GST occasioned by CP may predispose the liver to oxidative stress and tissue injury. In this study, pre-treatment and co-treatment with GA improved significantly the overall redox and antioxidant status in the liver of the rats. This observation is supported by previous findings [17,32,62,77]

Lipid peroxidation induced by free radical has been suggested to alter membrane structure and function and it is also implicated in cellular abnormalities such as mutation and cell death [78]. Increase in LPO is therefore a reliable marker to show index of oxidative stress and tissue damage [79]. The significant increase in hepatic lipid peroxidation as evidenced by the increased level of malondialdehyde (MDA) level in CP treated rats in this study indicates the involvement of free radical-induced oxidative cell injury in mediating the toxicity of cyclophosphamide. The attenuation of hepatic LPO by pre-treatment and co-treatment with gallic acid in this study may be due to free radical scavenging potential of the gallic acid. Gallic acid has been previously reported to protect against lipid peroxidation induced by CCl_4_ [32] and paracetamol [62].

## 5. Conclusions

This study clearly demonstrated the potential of gallic acid to offer protection against cyclophosphamide-induced organ toxicity and oxidative stress. Generally, gallic acid pre-treatment offered better antioxidant protection than co-administration with cyclophosphamide. We therefore suggest its possible use in chemotherapy and other stress-associated disorders as a supplementary/auxiliary therapy. However, further studies are required to clarify any potential interaction of gallic acid with the chemotherapeutic activity of cyclophosphamide.

## References

[1] Takimoto C.H., Calvo E., Pazdur R., Coia L.R., Hoskins W.J., Wagman L.D. (2005). Principles of oncologic pharmacotherapy. Cancer Management: A Multidisciplinary Approach.

[2] Clern M., Bickers D.R., Goodman G.A., Rall T.W., Nies A.S., Taylor P. (1991). Dermatological pharmacology. The Pharmacological Basis of Therapeutics.

[3] Paul C., Bruce A.C., Goodman G.A., Rall T.W., Nies A.S., Taylor P. (1991). Antineoplastic agents. The Pharmacological Basis of Therapeutics.

[4] Kirkland R.T., Bongiovanni A.M., Cornfield D., McCormic J.B., Parks J.S., Tenore A. (1976). Gonadotropin responses to leutinizing releasing factor in boys treated with cyclophosphamide for nephrotic syndrome. J. Pediatr..

[5] Etteldorf J.N., West C.D., Pitcock J.A., Williams D.L. (1976). Gonadal function, testicular histology, and meiosis following cyclophosphamide therapy in patients with nephrotic syndrome. J. Pediatr..

[6] Khan T.S., Sundin A., Juhlin C., Wilander E., Oberg K., Eriksson B. (2004). Vincristine, cisplatin, teniposide, and cyclophosphamide combination in the treatment of recurrent or metostatic adrenocortical cancer. Med. Oncol..

[7] Shanafelt T.D., Lin T., Geyer S.M., Zent C.S., Leung N., Kabat B., Bowen D., Grever M.R., Byrd J.C., Kay N.E. (2007). Pentostatin, cyclophosphamide, and rituximab regimen in older patients with chronic lymphocytic leukemia. Cancer.

[8] De Jonge M., Huitema A., Holtkamp M., van Dam S., Beijnea J., Rodenhuis S. (2005). Aprepitant inhibits cyclophosphamide bioactivation and thiotepa metabolism. Cancer Chemother. Pharmacol.

[9] Mcdonald G., Slattery J., Bouvier E., Song R., Batchelder A., Kalhorn T., Schoch H., Anasetti C., Goley T. (2003). Cyclophosphamide metabolism, liver toxicity, and mortality following hematopoietic stem cell transplantation. Blood.

[10] Hanane A., Claire L., Caroline A., Fabrice M., Paxton J. (2012). Anticancer Drug Metabolism: Chemotherapy Resistance and New Therapeutic Approaches. Topics on Drug Metabolism.

[11] Maiti A.K. (2012). Genetic determinants of oxidative stress-mediated sensitization of drug resistant cancer cells. Int. J. Cancer.

[12] Stankiewicz A., Skrzydlewska E., Makieła M. (2002). Effects of amifostine on liver oxidative stress caused by cyclophosphamide administration to rats. Drug Metabol. Drug Interact..

[13] Kern J.C., Kehrer J.P. (2002). Acrolein-induced cell death. A caspase-influenced decision between apoptosis and oncosis/necrosis. Chem Biol Interact..

[14] Premkumar K., Pachiappan A., Abraham S.K., Santhiya S.T., Gopinath P.M., Ramesh A. (2001). Effect of *Spirulina fusiformis* on cyclophosphamide and mitomycin-C induced genotoxicity and oxidative stress in mice. Fitoterapia.

[15] Venkatesan N., Chandrakasan G. (1994). *In vivo* administration of taurine and niacin modulate cyclophosphamide-induced lung injury. Eur. J. Pharmacol..

[16] Abariku S.O., Otuechere C.A., Eko M., Monwuba K., Osobu D. (2012). Rutin Ameliorates Cyclophosphamide-induced Reproductive Toxicity in Male Rats. Toxicol. Int..

[17] Premila A., Indirani K., Preethi K. (2008). Alterations in antioxidant enzyme activities and increased oxidative stress in cyclophosphamide-induced hemorrhagic cystitis in the rat. Cancer Ther..

[18] Selvakumar E., Prahalathan C., Mythili Y., Varalakshmi P. (2004). Protective effect of dl-β-lipoic acid in cyclophosphamide induced oxidative injury in rat testis. Reprod. Toxicol..

[19] Das U.B., Mallick M., Debnath J.M., Ghosh D. (2002). Protective effect of ascorbic acid on cyclophosphamide-induced testicular gametogenic and androgenic disorders in male rats. Asian J. Androl..

[20] McDermott E.M., Powell R.J. (1996). Incidence of ovarian failure in systemic lupus erythematosus after treatment with pulse cyclophosphamide. Ann. Rheum. Dis..

[21] Ghosh S., Ghosh D., Chattopadhyay S., Debnath J. (1999). Effect of ascorbic acid supplementation on liver and kidney toxicity in cyclophosphamide-treated female albino rats. J. Toxicol. Sci..

[22] Lee S., Schmitt C.A. (2003). Chemotherapy response and resistance. Curr. Opin. Genet. Dev..

[23] Weiji N.I., Cleton F.J., Osanto S. (1997). Free radicals and antioxidants in chemotherapy-induced toxicity. Cancer Treat. Rev..

[24] McCall M.R., Frei B. (1999). Can antioxidant vitamins materially reduce oxidative damage in humans?. Free Radic. Biol. Med..

[25] Singleton V.L. (1981). Naturally occurring food toxicants: Phenolic substances of plant origin common in foods. Adv. Food Res..

[26] Niu X., Fan X., Sun J., Ting P., Narula S., Lundell D. (2004). Inhibition of Fucosyltransferase VII by gallic acid and its derivative. Arch. Biochem. Biophys..

[27] Kim J.H., Lee B.K., Lee K.W., Lee H.J. (2009). Resveratrol Counteracts gallic acid-induced down regulation of gap-junction intercellular communication. J. Nutr. Biochem..

[28] Stanely P., Prince M., Priscilla H., Devika P.T. (2009). Gallic acid prevents lysosomal damage in isoproterenol induced cardiotoxicity in Wistar Rats. Eur. J. Pharmacol..

[29] Manach C., Scalbert A., Morand C., Remesy C., Jimenez L. (2004). Polyphenols: Food Sources and bioavailability. Am. J. Clin. Nutr..

[30] Priscilla D.H., Prince P.S. (2009). Cardioprotective effect of gallic acid on cardiac troponin-T, Cardiac marker enzymes, lipid peroxidation products and antioxidants in experimentally induced myocardial infarction in Wistar rats. Chem. Biol. Interact..

[31] Leiro I.M., Alvarez E., Arranz J.A., Siso I.G., Orallo F. (2014). *In vitro* effect of magniferin on superoxide concentration and expression of the inducible nitric oxide synthase, tumuor necrosis factor-alpha and transforming growth factor-beta genes. Biochem. Pharmacol..

[32] Mahmoud R.H., Barakat H. (2010). The protective effect of gallic acid and caffeine against CCl_4_-induced oxidative hepatotoxicity and mitochondrial DNA depletion in male albino rats. Egypt. J. Biochem. Mol. Biol..

[33] Padma V.V., Castro S.A., Felix T.A., Rathinasamy B., Paramasivan P. (2011). Protective effect of gallic acid against lindane induced toxicity in experimental rats. Food Chem. Toxicol..

[34] Kulkarni J.M., Viswanatha Swamy A.H.M. (2015). Cardioprotective effect of gallic acid against doxorubicin-induced myocardial toxicity in albino rats. Indian J. Health Sci..

[35] National Research Council (2011). Guide for the Care and Use of Laboratory Animals.

[36] Charan J., Kantharia N.D. (2013). How to calculate sample size in animal studies?. J. Pharmacol. Pharmacother..

[37] Punithavathi V.R., Prince P.S., Kumar R., Selvakumari J. (2011). Antihyperglycaemic, antilipid peroxidative and antioxidant effects of gallic acid on streptozotocin induced diabetic Wistar rats. Eur. J. Pharmacol..

[38] Jaffe B. (1972). What made the radical break. N. Engl. J. Med..

[39] Tietz N.W., Pruden E.L., Siggaard-Andersen O., Burtis A.C., Ashwood E.R. (1994). Liver function. Tietz Textbook of Clinical Chemistry.

[40] Tietz N.W., Tietz N.W. (1995). Clinical Guide to Laboratory Tests.

[41] Horder M., Magid E., Pitkanen E., Härkönen M., Strömme J.H., Theodorsen L., Gerhardt W., Waldenström J. (1979). Recommended method for the determination of creatine kinase in blood modified by the inclusion of EDTA. The committee on enzymes of the Scandinavian Society for Clinical Chemistry and Clinical Physiology (SCE). Scand. J. Clin. Lab. Investig..

[42] Erel O., Kocyigit A., Avci S., Aktepe N., Bulut V. (1997). Oxidative stress and antioxidative status of plasma and erythrocytes in patients with vivax malaria. Clin. Biochem..

[43] Jollow D.J., Mitchell J.R., Zampaglione N., Gillette J.R. (1974). Bromobenzene induced liver necrosis: Protective role of glutathione and evidence for 3,4-bromobenzene oxide as the hepatotoxic metabolite. Pharmacology.

[44] Varshney R., Kale R.K. (1990). Effects of calmodulin antagonists on radiation-induced lipid peroxidation in microsomes. Int. J. Radiat. Biol..

[45] Misra H.P., Fridovich I. (1972). The role of superoxide anion in the autoxidation of epinephrine and a simple assay for superoxide dismutase. J. Biol. Chem..

[46] Magwere T., Naik Y.S., Hasler J.A. (1996). Effects of chloroquine treatment on antioxidant enzymes in rat liver and kidney. Free Radic. Biol. Med..

[47] Sinha A.K. (1972). Colorimetric assay of catalase. Anal. Biochem..

[48] Habig W.H., Pabst M.J., Jakoby W.B. (1974). Glutathione transferases, the first enzymatic step in mercapturic acid formation. J. Biol. Chem..

[49] Lowry O.H., Rosebrough N.J., Farr A.L., Randall R.J. (1951). Protein measurement with the Folin phenol reagent. J. Biol. Chem..

[50] De Jonge M.E., Huitema A.D.R., Rodenhuis S., Beijnen J.H. (2005). Clinical Pharmacokinetics of cyclophosphamide. Clin. Pharmacokinet..

[51] Sangeetha P., Das U.N., Koratkar R., Suryaprabha P. (1990). Increase in free radical generation and lipid peroxidation following chemotherapy in patients with cancer. Free Radic. Biol. Med..

[52] Pratheeshkumar P., Kuttan G. (2010). *Cardiospermum halicacabum* inhibits cyclophosphamide induced immunosupression and oxidative stress in mice and also regulates *iNOS* and *COX-2* gene expression in LPS stimulated macrophages. Asian Pac. J. Cancer Prev..

[53] Jakopič J., Veberič R., Štampar F. (2009). Extraction of phenolic compounds from green walnut fruits in different solvents. Acta agriculturae Slovenica.

[54] Amacher D.E. (1998). Serum Transaminase Elevations as Indicators of Hepatic Injury Following the Administration of Drugs. Regulat. Toxicol. Pharmacol..

[55] Kumar G., Banu S.G., Kannan V., Pandian R.M. (2005). Antihepatotoxic effect of β-carotene on paracetamol induced hepatic damage in rats. Ind. J. Exp. Biol..

[56] Kaur G., Alam M.S., Jabbar Z., Javed K., Athar M. (2006). Evaluation of antioxidant activity of *Cassia siamea* flowers. J. Ethnopharmacol..

[57] Sreetha S., Padma P.R., Umadevi M. (2009). Effect of Coriandrum sativum extracts on carbon tetrachloride-induced hepatotoxicity in rats. Food Chem. Toxicol..

[58] Habibi E., Shokrzadeh M., Chabra A., Naghshvar F., Keshavarz-Maleki R., Ahmadi A. (2015). Protective effects of *Origanum vulgare* ethanol extract against cyclophosphamide-induced liver toxicity in mice. Pharm. Bio..

[59] Nyblom H., Bjornsson E., Simren M., Aldenborg F., Almer S., Olsson R. (2006). The AST/ALT ratio as an indicator of cirrhosis in patients with PBC. Liver Int..

[60] Singh A., Bhat T.K., Sharma O.P. (2011). Clinical Biochemistry of Hepatotoxicity. J. Clin. Toxicol..

[61] Ramaiah S.K. (2007). A toxicologist guide to the diagnostic interpretation of hepatic biochemical parameters. Food Chem. Toxicol..

[62] Rasool M.K., Sabina E.P., Ramya S.R., Preety P., Patel S., Mandal N., Mishra P.P., Samuel J. (2010). Hepatoprotective and antioxidant effect of gallic acid in paracetamol-induced liver damage in mice. J. Pharm. Pharmacol..

[63] Daugirdas J. (2011). CKD Screening and Management Overview. Handbook of Chronic Kidney Disease Management.

[64] Ferguson M.A., Waikar S.S. (2012). Established and Emerging Markers of Kidney Function. Clin. Chem..

[65] Gross J.L., de Azevedo M.J., Silveiro S.P., Canani L.H., Caramori M.L., Zelmanovitz T. (2005). Diabetic nephropathy: Diagnosis, prevention and treatment. Diabetes Care.

[66] Mouton R., Holder K. (2006). Laboratory tests of renal function. Anaesth. Intensive Care Med..

[67] Yamamoto T., Kikkawa R., Yamada H., Horii I. (2005). Identification of oxidative stress related proteins for predictive screening of hepatotoxicity using a proteomic approach. J. Toxicol. Sci..

[68] Oh J.M., Jung Y.S., Jeon B.S., Yoon B.I.I., Lee K.S., Kim B.H., Oh S.J., Kim S.K. (2012). Evaluation of hepatotoxicity and oxidative stress in rats treated with *tert-*butyl hydroperoxide. Food Chem. Toxicol..

[69] King P.D., Perry M.C. (2001). Hepatotoxicity of Chemotherapy. Oncolgist.

[70] Yost G.S., Horstman M.G., Walily A.F., Gordon W.P., Nelson S.D. (1985). Procarbazine spermatogenesis toxicity: Deuterium isotope effect point to regioselective metabolism in mice. Toxicol. Appl. Pharmacol..

[71] Valko M., Leibfritz D., Moncola J., Cronin M.T.D., Mazura M., Telser J. (2007). Free radicals and antioxidants in normal physiological functions and human disease. Int. J. Biochem. Cell Biol..

[72] Chance B., Sies H., Boveris A. (1979). Hydroperoxide metabolism in mammalian organs. Physiol. Rev..

[73] Han W.K., Bonventre J.V. (2004). Biological markers for the early detection of acute kidney injury. Curr. Opin. Crit. Care.

[74] Ali-Osman F. (1989). Quenching of DNA cross-link precursors of chloroethylnitrosoureas and attenuation of DNA interstrand cross-linking by glutathione. Cancer Res..

[75] Masella R., Di Benedetto R., Vari R., Filesi C., Giovannini C. (2005). Novel mechanisms of natural antioxidant compounds in biological systems: Involvement of glutathione and glutathione-related enzymes. J. Nutr. Biochem..

[76] Touliatos J.S., Neitzel L., Whitworth C., Rybak L.P., Malafa M. (2000). Effect of cisplatin on the expression of glutathione-*S*-transferase in the cochlea of the rat. Eur. Arch. Otorhinolaryngol..

[77] Pramita C., Ugir H.S.K., Nabendu M., Jayanta K.D., Smarajit P., Sudin B. (2009). Modulation of Cyclophosphamide-Induced Cellular Toxicity by Diphenylmethyl Selenocyanate *in vivo*, an Enzymatic Study. J. Cancer Mol..

[78] Senthil-Kumar M., Udayakumar M. (2006). High-throughput virus-induced gene-silencing approach to assess the functional relevance of a moisture stress-induced cDNA homologous to lea4. J. Exp. Bot..

[79] Gutteridge J.M. (1995). Lipid peroxidation and antioxidants as biomarkers of tissue damage. Clin. Chem..

